# Improving the Readability of Institutional Heart Failure–Related Patient Education Materials Using GPT-4: Observational Study

**DOI:** 10.2196/68817

**Published:** 2025-07-08

**Authors:** Ryan C King, Jamil S Samaan, Joseph Haquang, Vishnu Bharani, Samuel Margolis, Nitin Srinivasan, Yuxin Peng, Yee Hui Yeo, Roxana Ghashghaei

**Affiliations:** 1 Department of Medicine, Division of Cardiology, University of California, Irvine Medical Center, 101 The City Dr S, Orange, CA, 92868, United States, 1 714-456-7890; 2 Department of Medicine, Karsh Division of Gastroenterology and Hepatology, Cedars-Sinai Medical Center, Los Angeles, CA, United States; 3 David Geffen School of Medicine, University of California, Los Angeles, Los Angeles, CA, United States; 4 Keck School of Medicine, University of Southern California, Los Angeles, CA, United States; 5 School of Mathematics and Statistics, Xi'an Jiaotong University, Xi'an, China

**Keywords:** patient education, heart failure, artificial intelligence, large language models, ChatGPT, GPT-4, health literacy, readability

## Abstract

**Background:**

Heart failure management involves comprehensive lifestyle modifications such as daily weights, fluid and sodium restriction, and blood pressure monitoring, placing additional responsibility on patients and caregivers, with successful adherence often requiring extensive counseling and understandable patient education materials (PEMs). Prior research has shown PEMs related to cardiovascular disease often exceed the American Medical Association’s fifth- to sixth-grade recommended reading level. The large language model (LLM) ChatGPT may be a useful tool for improving PEM readability.

**Objective:**

We aim to assess the readability of heart failure–related PEMs from prominent cardiology institutions and evaluate GPT-4’s ability to improve these metrics while maintaining accuracy and comprehensiveness.

**Methods:**

A total of 143 heart failure–related PEMs were collected from the websites of the top 10 institutions listed on the 2022‐2023 US News & World Report for “Best Hospitals for Cardiology, Heart & Vascular Surgery.” PEMs were individually entered into GPT-4 (version updated July 20, 2023), preceded by the prompt, “Please explain the following in simpler terms.” Readability was assessed using the Flesch Reading Ease score, Flesch-Kincaid Grade Level (FKGL), Gunning Fog Index, Coleman-Liau Index, Simple Measure of Gobbledygook Index, and Automated Readability Index. The accuracy and comprehensiveness of revised GPT-4 PEMs were assessed by a board-certified cardiologist.

**Results:**

For 143 institutional heart failure–related PEMs analyzed, the median FKGL was 10.3 (IQR 7.9-13.1; high school sophomore) compared to 7.3 (IQR 6.1-8.5; seventh grade) for GPT-4’s revised PEMs (*P*<.001). Of the 143 institutional PEMs, there were 13 (9.1%) below the sixth-grade reading level, which improved to 33 (23.1%) after revision by GPT-4 (*P*<.001). No revised GPT-4 PEMs were graded as less accurate or less comprehensive compared to institutional PEMs. A total of 33 (23.1%) GPT-4 PEMs were graded as more comprehensive.

**Conclusions:**

GPT-4 significantly improved the readability of institutional heart failure–related PEMs. The model may be a promising adjunct resource in addition to care provided by a licensed health care professional for patients living with heart failure. Further rigorous testing and validation is needed to investigate its safety, efficacy, and impact on patient health literacy.

## Introduction

Heart failure affects approximately 1%‐2% of adults globally, with an estimated prevalence of 64 million people [1]. Treatment involves extensive patient adherence to lifestyle modifications such as daily weights, fluid and sodium restriction, and rigorous guideline-directed medication regimens. Altogether, these interventions attempt to prevent disease progression and hospital admissions, which drive most of the financial burden ($39.2-$60 billion) related to the disease [2]. Due to the complex degree of self-management required by patients with heart failure, improving patient education and health literacy may play a crucial role in improving outcomes [3,4].

In the United States, the average adult’s reading comprehension level is approximately seventh to eighth grade proficiency [5], resulting in the American Medical Association (AMA) recommendation of written patient education materials (PEMs) being at a fifth- to sixth-grade reading level [6]. However, a 2019 readability analysis of cardiovascular disease–related PEMs reported that the mean reading level of materials was tenth grade, comparable to that of a high school sophomore [7]. Inadequate health literacy has been associated with increased relative risk of emergency department visits, hospitalizations, and mortality for patients with heart failure [4,8], highlighting the need for accessible, readable, and high-quality PEMs.

ChatGPT is a large language model (LLM) that is gaining widespread public adoption [9]. With an increasing number of patients seeking health information online [10], the model has the potential to enhance patient health education and address the complexity of heart failure–related PEMs. As ChatGPT’s acceptance and usage have increased, initial research involved evaluating the model’s accuracy and reliability. Several studies have shown that ChatGPT provides appropriate, accurate, and reliable knowledge across a wide range of cardiac and noncardiac medical conditions, including heart failure [11-16]. In addition to accuracy, ChatGPT has been found to deliver more empathetic responses to real-world patient questions than physicians in online forums [17]. As prior data regarding accuracy have been promising, an emerging focus has been on investigating the readability of the model’s output.

Prior studies have shown ChatGPT provides accurate and comprehensive responses to questions related to heart failure, and another demonstrated its responses were at a college reading level, highlighting the need for further assessment of the readability of GPT’s outputs [12,18]. Similarly, another study examining GPT-4’s responses related to amyloidosis showed that while responses were often accurate and comprehensive, the average readability of responses ranged from a grade level of 10.3 (high school sophomore) to 21.7 (beyond graduate school) [16]. We aim to expand on the previous literature by assessing the readability of heart failure–related online PEMs from renowned cardiology institutions, assessing GPT-4’s ability to improve the readability of these PEMs, and comparing the accuracy and comprehensiveness between institutional PEMs and GPT-4’s revised PEMs.

## Methods

### Institutional Patient Education Materials

**Figure 1.**
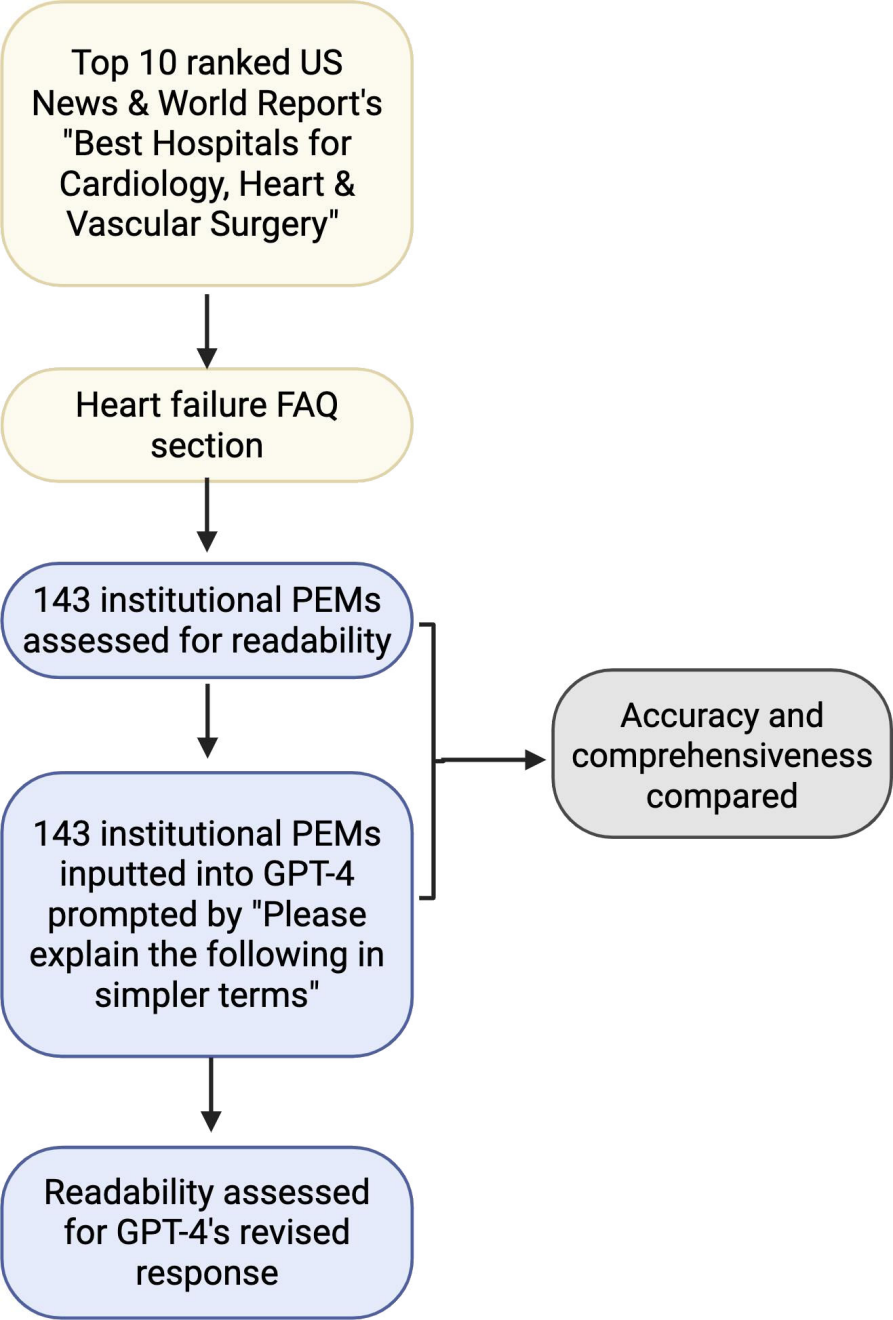
Diagram of institutional heart failure–related PEM curation, revised GPT-4 PEM generation, and subsequent assessment of readability, accuracy, and comprehensiveness. Created in BioRender [19]. FAQ: frequently asked question; PEM: patient education material.

There were 143 PEMs (Multimedia Appendix 1 and Figure 1) related to heart failure collected in July 2023 from the top 10 ranked cardiology institutions (deidentified) listed on the 2022‐2023 US News & World Report website as “Best Hospitals for Cardiology, Heart & Vascular Surgery.” These PEMs include frequently asked questions (FAQs) presented as text descriptions of various aspects of heart failure such as causes, symptoms, medications, and procedures. Duplicate institutional PEMs were included since education materials varied between institutions, and readability of each PEM was the primary outcome of interest.

### GPT-4 Response Generation

Each institution’s PEMs were entered into GPT-4 (version updated July 20, 2023), preceded by the prompt, “Please explain the following in simpler terms.” GPT-4 was accessed using the OpenAI website interface. Default model settings were used (temperature, max tokens, etc). The “new chat” function was used for each PEM, thus creating a new conversation without a record of prior inputs. Materials containing nontext components (images or videos) were excluded.

### Readability Assessment

The readability of institutional PEMs and GPT-4’s revised PEMs were then assessed using the following validated formulas: Flesch Reading Ease (FRE) score [20], Flesch-Kincaid Grade Level (FKGL) [21], Gunning Fog Index [22], Coleman-Liau Index [23], Simple Measure of Gobbledygook (SMOG) Index [24], and Automated Readability Index [25]. The FRE score, measured on a scale of 0 to 100, indicates a text with a higher score has better ease of understanding. The remaining formulas directly translate a score into its corresponding US reading grade level, such as a score of 10 translating to a tenth-grade reading level. These metrics derive their scores from the mean length of sentences and words used in a given text. In contrast to the FRE, lower scores in the other formulas correspond to an easier level of understanding. The readability formulas were assessed using the *Textstat* library in Python (Python Software Foundation) and the *Textstat readability* package in R software (R Foundation for Statistical Computing).

### Accuracy and Comprehensiveness

Accuracy and comprehensiveness of GPT-4’s revised PEMs (Multimedia Appendix 1) were assessed as secondary outcomes by an actively practicing board-certified cardiologist at a tertiary academic medical center. The reviewer was not blinded during grading. The reviewer used the following grading scale in Textbox 1 when grading the original institutional PEMs and revised GPT-4 PEMs.

Textbox 1. **Grading scale used by reviewer.**

“Compared to the institutional PEM, the GPT-4 revised PEM is”:

1. Less accurate
2. Equally accurate
3. More accurate

“Compared to the institutional PEM, the GPT-4 revised PEM is”:

1. Less comprehensive
2. Equally comprehensiveness
3. More comprehensive

### Statistical Analysis

Descriptive statistics are presented as medians and IQRs. Readability metrics for institutional PEMs and GPT-4’s revised PEMs were compared using the Mann-Whitney *U* test. Further subanalysis was performed investigating the proportion of PEMs meeting the sixth-grade reading level recommendation by the AMA among institutional PEMs and GPT-4’s revised PEMs. Statistical analysis was conducted using SPSS (version 29; IBM Corporation).

### Ethical Considerations

The data collection process in this observational study did not involve patients and did not require the deidentification or protection of data. Therefore, no institutional review board approval was sought.

## Results

### Readability Assessment

**Figure 2.**
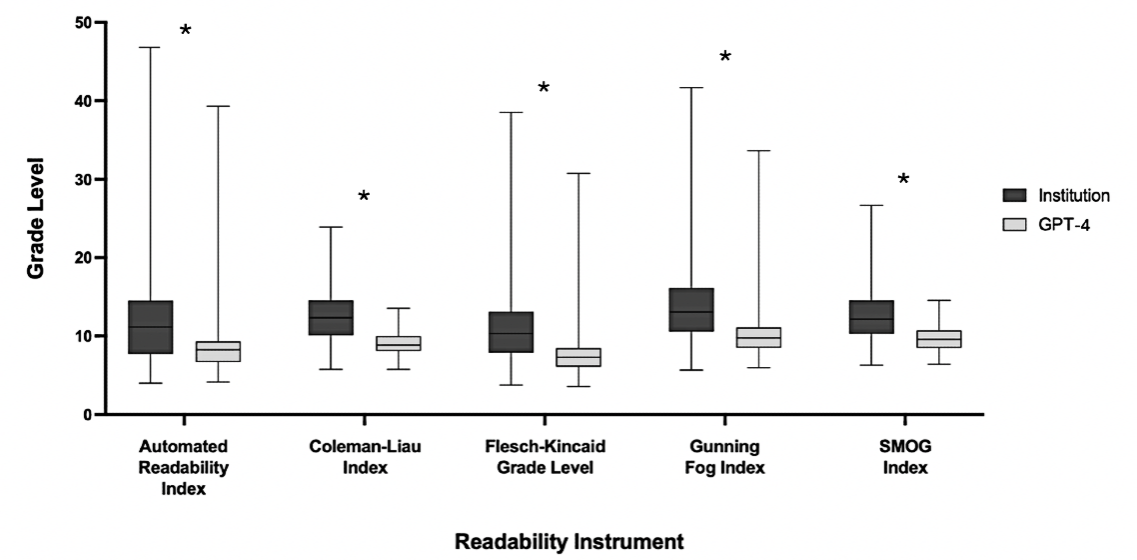
Box and whiskers plot of median readability scores across 5 metrics including Automated Readability Index, Coleman-Liau Index, Flesch-Kincaid Grade Level, Gunning Fog Index, Simple Measure of Gobbledygook (SMOG) Index for institutional and GPT-4’s revised PEMs. PEMs: patient education materials. * *P*<.05.

Readability analysis revealed GPT-4’s revised PEMs were significantly more readable compared to institutional PEMs across all 6 metrics (*P*<.001) (Figure 2). The FRE score increased from a median institutional score of 48.6 (IQR 38.0-63.3; *P*<.001; hard-to-read text, college reading level) to 72.2 (IQR 66.2-77.5; *P*<.001; fairly easy-to-read text, seventh-grade level) after GPT-4 revision [20]. The FKGL also saw improvement, decreasing from an institutional median reading level of tenth grade (IQR 7.9-13.1; *P*<.001) to seventh grade (IQR 6.1-8.5; *P*<.001) after GPT-4 revision. Furthermore, the institutional Automated Readability Index of 11.2 (IQR 7.7-14.5; *P*<.001) improved to 8.3 (IQR 6.7-9.3; *P*<.001) after GPT-4 revision. The other readability metrics (Gunning Fog Index, Coleman-Liau Index, and SMOG Index) also showed improved scores after GPT-4 revision: 9.8 (IQR 8.5-11.1; *P*<.001), 8.9 (IQR 8.1-10.0; *P*<.001), and 9.6 (IQR 8.5-10.7; *P*<.001), respectively, compared to the median institutional scores of 13.1 (IQR 10.6-16.2), 12.3 (IQR 10.1-14.5), and 12.2 (IQR 10.3-14.6). Before GPT-4 revision, 9.1% (13/143) of institutional PEMs met the AMA’s recommended sixth-grade reading level (Table 1). However, after GPT-4’s revision, 23.1% (33/143) of PEMs met the sixth-grade recommendation. On average, GPT-4 revision led to a 3.6 reading grade level reduction.

An example of this simplification in reading level was seen when describing different types of heart failure. The institutional PEM described right-sided heart failure as most often resulting from left-sided heart failure due to increased pressure from the left ventricle not propelling blood to the rest of the body. However, GPT-4 provided a more basic explanation using an analogy of ventricles being small rooms and gave a more simplified explanation of right-sided heart failure as a result of left-sided heart failure. In another example, when explaining the various causes of heart failure, one institutional PEM provided a list of etiologies such as “heart valve disease” or “coronary artery disease” without a description, compared to GPT-4, which more thoroughly described the role of each cause in relation to heart failure in simple language.

**Table 1.** Comparison of the proportion of patient education materials (PEMs) meeting the American Medical Association’s (AMA) recommended sixth-grade reading level between institutional and GPT-4’s revised PEMs.

	≤Sixth-grade reading level	≥Sixth-grade reading level	Percent meeting AMA recommendation
Institutional Flesch-Kincaid Grade Level	13	130	9.10
GPT-4 Flesch-Kincaid Grade Level	33	110	23.10

### Accuracy and Comprehensiveness

Following review by a board-certified cardiologist, 33 out of 143 (23.1%) revised GPT-4 PEMs were graded as more comprehensive than the corresponding institutional PEMs (Table 2). Additionally, all 143 (100%) revised GPT-4 PEMs were graded as equally accurate as their institutional PEM counterpart.

**Table 2.** Evaluation of GPT-4’s accuracy and comprehensiveness of revised patient education materials (PEMs) compared to institutional PEMs (N=143).

Scoring	Accuracy, n (%)	Comprehensiveness, n (%)
Less	0 (0)	0 (0)
Equal	143 (100)	110 (76.9)
More	0 (0)	33 (23.1)

## Discussion

### Principal Results

LLMs are a rapidly developing technology with the potential to enhance the delivery of PEMs to patients of all levels of health literacy. In this study, we expanded on existing research that evaluated ChatGPT’s ability to generate accurate and reliable answers to heart failure questions by examining GPT-4’s ability to improve the readability of institutional PEMs. Our analysis shows that GPT-4, when prompted, was able to significantly enhance the readability of institutional PEMs for common heart failure–related patient questions. After evaluation by a board-certified cardiologist, all of GPT-4’s revised PEMs were graded as equally accurate and many were graded as more comprehensive as institutional PEMs, with no revised PEMs graded as less accurate or less comprehensive. GPT-4’s capabilities to provide accurate, comprehensive, and readable PEMs in real-time and in a conversational manner underscores the future potential of LLMs to enhance patient education and ultimately patient health literacy.

### Comparison With Prior Work

Previous research has demonstrated that ChatGPT possesses a broad knowledge base comprising various medical conditions, including cirrhosis, hepatocellular carcinoma, and bariatric surgery [14,15,26,27]. Its knowledge base also spans cardiovascular diseases such as acute coronary syndrome [11,28], heart failure [12], atrial fibrillation [29], and even rare disorders like amyloidosis [16]—a multisystemic infiltrative disease. Specifically, regarding amyloidosis, while GPT-4 provided accurate, comprehensive, and reliable answers to gastrointestinal, neurologic, and cardiology queries, the average FKGL of responses was 15.5 (college level), significantly exceeding the recommended sixth-grade reading level set forth by the AMA [16]. Similar results were shown when examining responses to the surgical treatment of retinal diseases and hypothyroidism in pregnancy [30,31].

A previous study examined ChatGPT’s ability to simplify the readability of responses to bariatric surgery–related FAQs [32]. GPT-4 reduced the average grade reading level of PEMs from eleventh (high school junior) to sixth grade, aligning with the AMA’s recommendation. Another study also showed that GPT-4 improved the readability of cardiovascular magnetic resonance reports, reducing the average reading level from tenth grade to fifth grade while maintaining high factual accuracy [33]. When simplifying PEMs relating to aortic stenosis, GPT-3.5 was able to lower the mean FKGL from 9.2 to 5.9 when instructed to “translate to a 5th grade reading level” [34]. Our study further contributes to this body of work by demonstrating GPT-4’s ability to improve the median readability of institutional PEMs from 10.3 (high school sophomore) to 7.3 (seventh grade) while maintaining accuracy and often enhancing comprehensiveness (Table 1). However, a unique aspect of our study was the use of a general prompt, “Please explain the following in simpler terms,” compared to other studies that specifically requested simplification to a fifth- to sixth-grade reading level [34]. Our prompt simulates an organic patient encounter with the GPT-4 platform written in language meant to mirror an actual patient request for simplification. This difference in prompting but similar significant improvement in readability shows the adaptability of LLMs in this domain and may increase the likelihood of future adoption. Furthermore, the enhanced readability underscores the potential of LLMs in fostering better patient understanding of heart failure–related information.

### Limitations and Ethical Concerns

ChatGPT, while adept at generating conversational answers, has inherent limitations in accuracy and privacy. The model cannot access real-time patient records and often does not cite peer-reviewed articles or reference updated guidelines, which is crucial for accurate and evidence-based responses. Additionally, the current model may not reliably understand nuanced medical topics or accurately interpret complex medical questions [35], leading to potential patient misunderstandings. In some cases, ChatGPT may also generate answers that initially seem factual due to its confident-appearing language but disseminate inaccurate information, known as artificial hallucinations [36]. Utilizing artificial intelligence (AI) models like ChatGPT in health care settings may also not guarantee secure handling of patient information as the model may collect users’ conversation data for future training. Although OpenAI does have a privacy setting allowing for disabling user data collection, prioritizing patient confidentiality will be an important aspect of development if the technology is to be used as an adjunct health care tool [37].

Furthermore, ChatGPT may also perpetuate social disparities due to implicit biases and contribute to accessibility gaps. Recent studies revealed that GPT-4 tended to promote outdated race-based medicine and overrepresent or underrepresent certain racial groups and sexes depending on the circumstance and thus potentially reinforce stereotypes [38,39]. Another concern is equitable access, as patients with lower socioeconomic status often have less access to certain technology such as the internet and may have barriers to utilizing these new AI tools [40]. Altogether, these validity and ethical considerations emphasize that clinical oversight, such as US Food and Drug Administration regulation, is warranted prior to LLM incorporation in patient care [41]. This would allow for consistent monitoring of this rapidly evolving technology, ensuring optimization of safety protocols with each new update of the model.

Our study has several limitations. Although we employed validated readability scoring systems as a surrogate for patient understanding, these formulas have their limitations, as previously reported [42,43]. These formulas often generate a reading level score that inherently grades longer words and sentences as being more complex but are unable to assess a text’s content for structure and clarity. Our study also did not involve patients, which is essential for the comprehensive assessment of ChatGPT as a patient educational resource. Future studies would benefit from involving patients to ensure relevance of questions, preference in language used, and assessment of patient understanding. A baseline assessment of a patient’s understanding of the given topic would also be beneficial to assess if ChatGPT can improve comprehension rather than relying on scoring tools. Additionally, we employed only one expert reviewer to assess the accuracy and comprehensiveness of ChatGPT’s responses. To limit the potential for bias through subjective review and promote diverse perspectives, future research would benefit from involving multiple reviewers from different backgrounds and training institutions. Our reviewer was also not blinded to the source of each PEM, allowing for possible bias when evaluating accuracy and comprehensiveness. Our study could also not incorporate or interpret questions containing multimedia at the time of data collection, but with the release of multimodal LLMs, like GPT-4v, including visual aids would be another valuable component of PEMs to investigate. The PEMs used are not comprehensive of all questions that may be asked by patients, which limits the generalizability of our results. Future studies using real-world patients and questions would be helpful to further understand the broad spectrum of questions patients may ask.

### Future Directions

We opted for a pragmatic approach in designing the GPT-4 prompt used to revise institutional PEMs. Our focus was on ensuring the prompt reflected a simple, intuitive command that patients would be likely to use in real-world scenarios. Although this method provided promising results, highlighting the versatility of GPT-4, exploring more intricate prompts may yield even more impressive outputs and functionality. We advocate further research into prompt engineering to better replicate natural conversations and offer specific instructions for generating higher-quality and personalized responses.

Medical institutions can utilize this technology by integrating ChatGPT directly into their online patient education platforms with customized readability based on the highest level of education completed by the patient. This type of personalization of readability assessment can be implemented in all patient-facing AI applications to ensure the appropriate reading level of text for all patients. For example, Buoy Health, a chatbot developed by Harvard Medical School in 2014, uses natural language processing to help users assess symptoms with reported accuracy rates of 90%‐98% [44,45]. Boston Children’s Hospital has adopted this platform on their website to guide patients on symptoms and recommended next steps in seeking medical care [44,45]. While not solely focused on education, it demonstrates how leading institutions are successfully leveraging chatbots as interactive tools. The consideration of readability assessment and adaptability in these patient-facing applications may increase patient engagement and ensure patients of all education levels can use these tools. Greater collaboration between trusted medical institutions and LLM platforms could improve patient access to simplified, accurate medical information that aligns with the AMAs recommended fifth- to sixth-grade reading level.

### Conclusions

Our study demonstrates GPT-4’s ability to improve the readability of institutional heart failure–related PEMs while also maintaining accuracy and comprehensiveness. Our results underscore the potential future utility of LLMs in improving the delivery of easy-to-understand and readable PEMs to patients of all health literacy levels. While ChatGPT may potentially be a valuable future tool in patient care, it should be used as a supplement to, rather than a replacement for, human expertise and judgment of a licensed health care professional. We recommend the development of future studies examining the optimization of readability outputs, personalization, and real-world implementation.

## Supplementary material

10.2196/68817 Multimedia Appendix 1 Accuracy and comprehensiveness data.

10.2196/68817 Multimedia Appendix 2 Comparison of readability of institutional and GPT-4’s revised patient education materials.

## Abbreviations

AI: artificial intelligence
AMA: American Medical Association
FAQ: frequently asked question
FKGL: Flesch-Kincaid Grade Level
FRE: Flesch Reading Ease score
LLM: large language model
PEM: patient education material
SMOG: Simple Measure of Gobbledygook
