# Supplementary figures and images for: Improving the Readability of Institutional Heart Failure–Related Patient Education Materials Using GPT-4: Observational Study
Source: JMIR Cardio. 2025 Jul 8;9:e68817. doi: 10.2196/68817 (PMC12263092; doi:10.2196/68817)

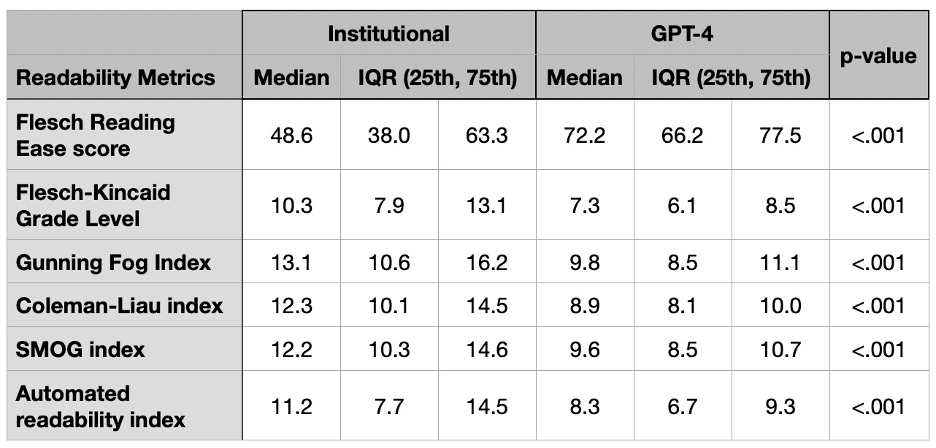

Supplement: Multimedia Appendix 2 [file cardio-v9-e68817-s002.png]
